# Using Ligand-Mapping Simulations to Design a Ligand Selectively Targeting a Cryptic Surface Pocket of Polo-Like Kinase 1**

**DOI:** 10.1002/anie.201205676

**Published:** 2012-09-07

**Authors:** Yaw Sing Tan, Paweł Śledź, Steffen Lang, Christopher J Stubbs, David R Spring, Chris Abell, Robert B Best

**Affiliations:** a Department of Chemistry, University of CambridgeLensfield Road, Cambridge, CB2 1EW (UK); b Present address: Department of Molecular Structural Biology, Max Planck Institute of BiochemistryAm Klopferspitz 18, 82152 Martinsried (Germany)

**Keywords:** drug design, molecular dynamics, molecular recognition, protein dynamics, protein–protein interactions

The treatment of protein flexibility is a major challenge in structure-based drug design (SBDD)^1^, ^2^ as proteins are dynamic and commonly undergo conformational changes to bind ligands.^3^–^6^ Consequently, binding sites may not be apparent in experimental structures of the unliganded protein. As a prototypical example, we focus here on the polo-box domain (PBD) of polo-like kinase 1 (Plk1), a serine/threonine kinase that is overexpressed in a wide range of cancers,^7^, ^8^ and is a known anticancer target due to its critical role in mitotic progression.^9^ The PBD helps in subcellular localization of the protein by binding to serine- or threonine-phosphorylated sequences at a polar phosphopeptide binding site (Figure 1 a).^10^, ^11^

**Figure 1:**
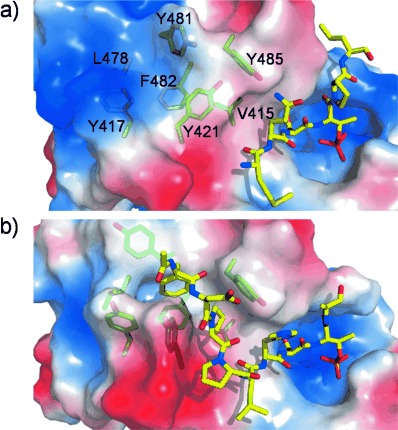
Binding pockets of Plk1 PBD as revealed by phosphopeptide ligands. Residues of the hydrophobic binding site are shown in green sticks and ligands in yellow sticks. Regions of negative, positive and neutral electrostatic potential on the protein surface are red, blue and white, respectively. a) Crystal structure of a phosphopeptide bound to the positively charged phosphopeptide binding pocket (PDB code 1Q4K), with closed hydrophobic pocket. b) Crystal structure of a phosphopeptide bound to both binding pockets (PDB code 3P37), with open hydrophobic pocket.

Recently, a secondary hydrophobic binding site (Figure 1 b) proximal to the primary phosphopeptide binding site has been identified by two independent approaches.^12^, ^13^ Crystal structures have revealed that this pocket can accommodate hydrophobic side-chains of several ligands in slightly different binding modes, with a resulting increase in affinity;^12^–^16^ however, when ligands are not bound to it, the pocket is closed. This cryptic pocket therefore presents a classic problem in SBDD targeting a flexible protein surface. Here, we show that although opening of the pocket is highly unfavorable in the absence of a ligand, it is possible to identify all of its known ligand-binding modes, as well as a novel mode, by using a modified ligand-mapping technique. The previously unknown binding mode was used as a basis for the design of a new ligand with similar affinity to others binding this pocket. The predictions were validated by solving the crystal structure of the bound complex.

The walls of the hydrophobic PBD secondary binding site comprise Tyr417, Tyr421, Leu478, Tyr481 and Tyr485, with Val415 and Phe482 lying at the bottom. Side-chain movements of Tyr417 and Tyr481 allow for the accommodation of a phenyl or other hydrophobic moiety. We first attempted to generate a conformational ensemble of the hydrophobic binding site by performing a 50 ns molecular dynamics (MD) simulation of the unliganded protein in explicit water, using the Amber ff99SB-ILDN force field.^17^, ^18^ This approach is similar to the relaxed complex scheme successfully used in other studies.^19^–^21^ Root-mean-square deviation(RMSD)-based clustering of the MD trajectory was performed to compare the conformations of the binding site with those seen before in various crystal structures. Four distinct closed conformations of the site were identified, three of which are also observed in crystal structures (Figure 2 a–d).

**Figure 2:**
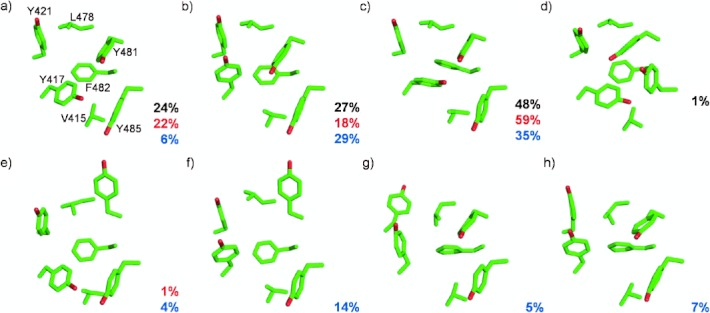
Conformations of the hydrophobic binding pocket in Plk1 PBD. Percentage populations of the conformations observed in the single long, multiple short and ligand-mapping simulations are indicated in black, red and blue, respectively. a–c) Closed conformations observed in crystal structures. d) Closed conformation observed only in the single long MD simulation. e,f) Open conformations observed in crystal structures. g,h) New conformations observed in ligand-mapping MD simulations.

In contrast, no conformations in which the key residue Tyr481 is “open” (e.g. Figure 2 e,f) were seen during the simulation. The high propensity of Tyr481 to adopt its closed conformation was confirmed by a second simulation of the unliganded PBD, starting from a crystal structure with the pocket open, after removing the ligand (PDB code 3P37). Tyr481 closed over the pocket after about 2 ns and remained so for the rest of the 50 ns simulation (see Supporting Information, Figure S1b). In an attempt to maximize the coverage of conformational space, we also ran 10 independent 5 ns simulations instead of a single long one.^22^ Opening of Tyr481 was observed in one of these runs, however the pocket did not fully open as Tyr417 remained in its closed conformation (Figure 2 e). When this simulation was extended, Tyr481 closed after 2 ns and did not reopen.

To gain more insight into the lack of pocket opening events, we estimated the free energy for opening by using umbrella sampling^23^ to bias the side-chain of Tyr481 to sample different *χ*1 torsion angle values. The free energy difference between its open and closed conformations was estimated to be ca. 4 kcal mol^−1^ with a barrier of ca. 6 kcal mol^−1^ (Figure S2). This explains the infrequent sampling of open conformations by Tyr481 within the timescale of the unliganded simulations, and its rapid closure in the absence of a stabilizing ligand.

These observations highlight the difficulties of using unbiased MD trajectories to identify cryptic binding sites when ligand-binding protein conformations are rarely sampled in the absence of a ligand: “solvent mapping” approaches, in which binding sites are identified by scanning model ligands over the protein surface,^24^ require the sites to be accessible. Methods such as replica exchange MD^25^ and metadynamics^26^ can improve sampling, but are not specific to opening hydrophobic pockets. Here, we adopt an alternative enhanced sampling scheme, suggested by the open conformations in crystal structures all being stabilized by either a hydrophobic ligand or crystal contact.^12^ We thus increased the sampling of relevant ligand-binding conformations by incorporating benzene molecules into the simulations, due to the pocket’s known affinity for a phenyl moiety.^12^, ^13^ Although hydrophilic molecules have previously been used for this purpose at high (2.7 m) concentrations,^27^ a lower concentration of 0.2 m was needed in this work to avoid phase separation of benzene and water. Note that an alternative method, site-identification by ligand competitive saturation (SILCS),^28^–^30^ uses high concentrations of hydrophobic ligands (1 m) in conjunction with an inter-ligand repulsive interaction energy term. However, SILCS’s main motivation is to characterize the protein surface through *competition* between ligands representative of different functionalities. Our aim is to identify only binding pockets with a high affinity for aromatic groups, in which case a lower benzene concentration is more selective. The absence of a repulsive potential also allows for accommodation of multiple benzene molecules in the same binding pocket—as observed in some of our simulations.

For optimal conformational sampling, 10 independent 5 ns ligand-mapping simulations were performed with different initial benzene distributions. All binding pocket conformations previously seen in crystal structures were reproduced by the simulations (Figure 2). The open pocket conformations were stabilized by the binding of benzene (Figure 3 a,b). Mapping of benzene occupancy for trajectory structures with similar benzene binding modes at the pocket correlated well with previously seen interactors of the pocket, showing that our ligand-mapping simulations reproduce crystallographic binding modes of ligands on the PBD surface (Figure 3 c,d). In addition, in some of the simulations, we observed “half-open” conformations of the pocket (Figure 2 g,h) not seen in crystal structures. These conformations are involved in a new “half open” binding mode in which a benzene molecule lodges in a smaller cleft formed by five of the hydrophobic pocket residues: Val415, Tyr417, Tyr481, Phe482 and Tyr485 (Figure 4 a).

**Figure 3:**
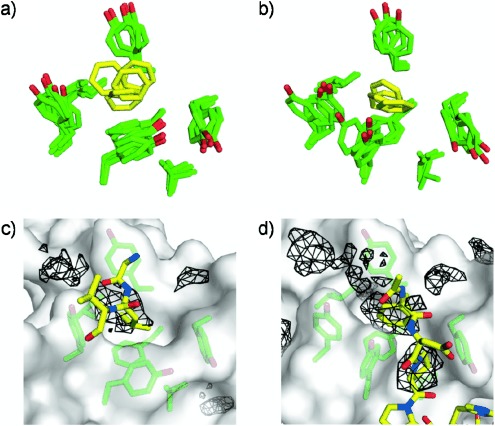
Reproduction of crystallographic binding modes at PBD hydrophobic pocket by ligand-mapping simulations. a,b) Superposition of five snapshots of the partially and fully open binding pocket, respectively, from ligand-mapping simulations. Pocket residues are green and benzene molecules are yellow. c) Benzene probes mimic the crystal-packing interaction of Leu394 from a neighboring protomer with the binding pocket (PDB code 3P35). Interacting ligands and crystal contact residues are shown in yellow sticks while regions sampled by benzene probes for this protein conformation are represented as black mesh (see Supporting Information for details). Only the part of the protomer binding to the pocket is shown. d) Benzene probes mimic the interactions of Phe and Pro from the peptide ligand with the binding pocket in the PBD/FDPPLHSpTA complex (PDB code 3P37).

**Figure 4:**
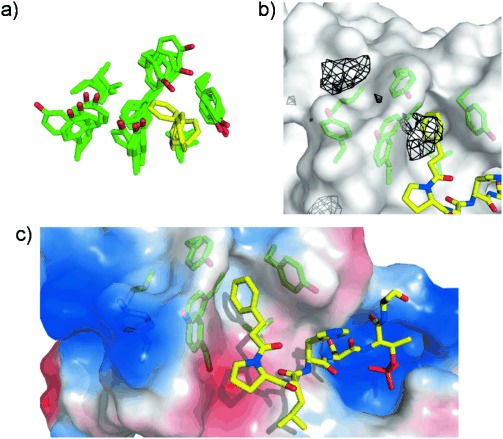
Design of a chimeric peptide ligand based on an alternative binding mode obtained from ligand-mapping simulations. a) Superposition of five snapshots of PBD binding pocket (green) from a ligand-mapping MD trajectory, showing an alternative binding mode of benzene (yellow). b) Binding mode of chimeric peptide (yellow sticks) at PBD pocket (green sticks) overlaid with benzene probability isosurfaces (black mesh) for the protein structure shown. c) Crystal structure of PBD complexed with chimeric peptide (yellow sticks). Regions of positive and negative electrostatic potential on the PBD surface are colored blue and red, respectively.

The generation of previously observed binding modes at the pocket in the ligand-mapping simulations is encouraging. To further validate the simulation results, we designed a ligand to present a phenyl ring to the pocket in the half-open conformation seen in the simulations (Figure 4 a). Based on the known structure of FDPPLHSpTA bound to PBD (Figure 1 b), the new ligand was modeled by replacing the N-terminal FDP residues with a 3-phenylpropanoyl moiety, which incorporates a minimal aliphatic linker between the phenyl group in the pocket and the truncated peptide. Evidence for the stability of this chimeric peptide ligand at the pocket was provided by an MD simulation of its complex with PBD (Figure S3, S4 and Table S1).

We synthesized the ligand and determined its *K*_D_ to be 330 nm by isothermal titration calorimetry (ITC), comparable to that of the significantly larger FDPPLHSpTA peptide (250 nm). A crystal structure of the complex (PDB code 4E67) was also obtained, showing that the binding mode of the new chimeric ligand at the chosen binding pocket is indeed similar to that envisaged in the design (Figure 4 b,c). This demonstrates specificity in our design, as other phosphopeptides are known to bind in a completely different mode, avoiding the pocket altogether.^12^

We have shown that the introduction of benzene probe molecules into MD simulations considerably expands the accessible conformational space of the flexible PBD hydrophobic pocket. We have also used this ligand-mapping approach for the first time to design a novel high-affinity ligand, whose binding mode was confirmed by X-ray crystallography. Simulations with explicit solvent are undoubtedly expensive for drug design. However, our results provide a proof of principle which may be used as the basis for more efficient sampling strategies in future, for example by using probe molecules in conjunction with an implicit water model.^31^, ^32^ In this work, we have focused on a hydrophobic pocket. However, there is no reason that a library of small molecules presenting different chemical functionalities could not be similarly used to expose other types of cryptic binding sites.

## Notes

Detailed facts of importance to specialist readers are published as ”Supporting Information”. Such documents are peer-reviewed, but not copy-edited or typeset. They are made available as submitted by the authors.

## References

[1] Cozzini P, Kellogg GE, Spyrakis F, Abraham DJ, Costantino G, Emerson A, Fanelli F, Gohlke H, Kuhn LA, Morris GM, Orozco M, Pertinhez TA, Rizzi M, Sotriffer CA (2008). J. Med. Chem.

[2] Fuentes G, Dastidar SG, Madhumalar A, Verma CS (2011). Drug Dev. Res.

[3] Betts MJ, Sternberg MJE (1999). Protein Eng.

[4] Withers IM, Mazanetz MP, Wang H, Fischer PM, Laughton CA (2008). J. Chem. Inf. Model.

[5] DeLano WL, Ultsch MH, de Vos AM, Wells JA (2000). Science.

[6] Teague SJ (2003). Nat. Rev. Drug Discovery.

[7] Holtrich U, Wolf G, Brauninger A, Karn T, Bohme B, Rubsamenwaigmann H, Strebhardt K (1994). Proc. Natl. Acad. Sci. USA.

[8] Lowery DM, Lim D, Yaffe MB (2005). Oncogene.

[9] Strebhardt K, Ullrich A (2006). Nat. Rev. Cancer.

[10] Song S, Grenfell TZ, Garfield S, Erikson RL, Lee KS (2000). Mol. Cell. Biol.

[11] Reynolds N, Ohkura H (2003). J. Cell Sci.

[12] Śledź P, Stubbs CJ, Lang S, Yang YQ, McKenzie GJ, Venkitaraman AR, Hyvonen M, Abell C (2011). Angew. Chem.

[13] Liu F, Park J-E, Qian W-J, Lim D, Graeber M, Berg T, Yaffe MB, Lee KS, Burke TR (2011). Nat. Chem. Biol.

[14] Liu F, Park J-E, Qian W-J, Lim D, Scharow A, Berg T, Yaffe MB, Lee KS, Burke TR (2012). ACS Chem. Biol.

[15] Liu F, Park J-E, Qian W-J, Lim D, Scharow A, Berg T, Yaffe MB, Lee KS, Burke TR (2012). ChemBioChem.

[16] Śledź P, Lang S, Stubbs CJ, Abell C (2012). Angew. Chem.

[17] Hornak V, Abel R, Okur A, Strockbine B, Roitberg A, Simmerling C (2006). Proteins Struct. Funct. Bioinf.

[18] Lindorff-Larsen K, Piana S, Palmo K, Maragakis P, Klepeis JL, Dror RO, Shaw DE (2010). Proteins Struct. Funct. Bioinf.

[19] Lin JH, Perryman AL, Schames JR, McCammon JA (2002). J. Am. Chem. Soc.

[20] Schames JR, Henchman RH, Siegel JS, Sotriffer CA, Ni HH, McCammon JA (2004). J. Med. Chem.

[21] Cheng LS, Amaro RE, Xu D, Li WW, Arzberger PW, McCammon JA (2008). J. Med. Chem.

[22] Caves LSD, Evanseck JD, Karplus M (1998). Protein Sci.

[23] Torrie GM, Valleau JP (1977). J. Comput. Phys.

[24] Miranker A, Karplus M (1991). Proteins Struct. Funct. Genet.

[25] Sugita Y, Okamoto Y (1999). Chem. Phys. Lett.

[26] Laio A, Parrinello M (2002). Proc. Natl. Acad. Sci. USA.

[27] Seco J, Luque FJ, Barril X (2009). J. Med. Chem.

[28] Guvench O, MacKerell AD (2009). PLoS Comp. Biol.

[29] Raman EP, Yu W, Guvench O, MacKerell AD (2011). J. Chem. Inf. Model.

[30] Foster TJ, Mackerell AD, Guvench O (2012). J. Comput. Chem.

[31] Haberthür U, Caflisch A (2008). J. Comput. Chem.

[32] Zoete V, Grosdidier A, Cuendet M, Michielin O (2010). J. Mol. Recognit.

