# Supplementary material for: Using Ligand-Mapping Simulations to Design a Ligand Selectively Targeting a Cryptic Surface Pocket of Polo-Like Kinase 1
Source: Angew Chem Int Ed Engl. 2012 Sep 7;51(40):10078–81. doi: 10.1002/anie.201205676 (PMC3547296; doi:10.1002/anie.201205676)
Supplement: Supplementary file 1 [file anie0051-10078-SD1.pdf]

Angewandte Chemie
Eine Zeitschrift der Gesellschaft Deutscher Chemiker

Supporting Information

© Wiley-VCH 2012

69451 Weinheim, Germany

Using Ligand-Mapping Simulations to Design a Ligand Selectively Targeting a Cryptic Surface Pocket of Polo-Like Kinase 1**

Yaw Sing Tan, Paweł Śledź, Steffen Lang, Christopher J. Stubbs, David R. Spring, Chris Abell, and Robert B. Best**

anie_201205676_sm_miscellaneous_information.pdf

Molecular dynamics simulations

Preparation of structures

The initial structures of polo-like kinase 1 (Plk1) polo-box domain (PBD) were obtained from the Protein Data Bank. One structure contains a peptide (LHSpTA) bound to the phosphopeptide pocket (PDB code 3FVH[1], resolved at 1.58 Å) and with the hydrophobic pocket in the closed state, while the other contains a peptide (FDPPLHSpTA) bound to both the phosphopeptide and open hydrophobic pockets (PDB code 3P37[2], resolved at 2.38 Å). Their peptides were removed to generate two unliganded PBD structures. Crystallographic water molecules were retained. PDB2PQR[3] was used to determine the protonation states of residues and to add missing hydrogen atoms.

Molecular dynamics

The antechamber and LEaP programs in the AMBER 11 package[4] were used to set up the systems for molecular dynamics (MD) simulation. Each system was neutralized with either sodium or chloride ions and solvated with TIP3P water molecules[5] in a periodic truncated octahedron box such that its walls were at least 9 Å away from the solute. In total, three PBD systems were set up: unliganded PBD with closed pocket, unliganded PBD with open pocket, and PBD complexed with FDPPLHSpTA peptide. Energy minimizations and MD simulations were performed with the sander and PMEMD modules of AMBER 11, using the ff99SB-ILDN[6-7] force field for the protein and peptide and the GAFF[8] force field for benzene. Parameters for phosphoserine in the -2 charge state were developed by Homeyer et al.[9] SHAKE[10] was applied to constrain all bonds involving hydrogen atoms, allowing for a time step of 2 fs. Nonbonded interactions were truncated at 9 Å and the particle mesh Ewald (PME)[11] method was used to

calculate long range electrostatic interactions under periodic boundary conditions, using a mesh spacing of 1.0 Å. With positional restraints on the solute atoms, 500 cycles of steepest descent and 500 cycles of conjugate gradient energy minimizations were carried out followed by two 50-ps MD equilibration runs: in the first equilibration run, the system was heated gradually from 0.1 to 300K at constant volume while in the second equilibration run, the system was at a constant pressure of 1 atm and temperature of 300 K. Subsequent unrestrained equilibration (2 ns) and production (50 ns) runs were carried out at constant temperature (300 K) using a Langevin thermostat[12] with a collision frequency of 2 ps^{-1} and constant pressure (1 atm) using a Berendsen barostat[13] with a pressure relaxation time of 2 ps. The 10 independent 5-ns MD simulations of unliganded PBD with closed pocket were initiated with different atomic velocities and seeds for the pseudorandom number generator and equilibrated as described above.

Umbrella sampling

Umbrella sampling[14] was performed to obtain the free energy profile for the $\chi 1$ side chain dihedral of Y481. The fully equilibrated structure of unliganded PBD with closed pocket was used as the initial structure for all the umbrella sampling simulations. Thirteen 3-ns simulations were performed by varying the favoured angle from -180° to 180° in increments of 30°. The torsion angle restraint used was 15 kcal mol^{-1} rad^{-2}. Positional restraints were placed on all atoms except for those belonging to Y481, Y417 and F482 in order to reduce the need to equilibrate over many slow orthogonal degrees of freedom. The combined results from the last 2 ns of the simulations were analyzed using the weighted histogram analysis method (WHAM).[15] We have assessed the convergence of the simulation sampling by comparing the PMF calculated using the time range 1-2 ns and 1-3 ns, and we obtain very similar results (Figure S2).

Ligand-mapping MD simulations

The initial unliganded PBD structure for ligand-mapping simulations was derived from the Protein Data Bank (PDB code 3FVH) as described above. Packmol[16] was used to generate 10 different placements of 40 benzene molecules within 40 Å of the protein centre. Each of the 10 systems was neutralized with a chloride ion and solvated with TIP3P water molecules in a periodic truncated octahedron box to yield a concentration of ~0.2 M benzene. Minimization, equilibration and production (5 ns) MD simulations were carried out as described above, for a cumulative sampling time of 50 ns.

Ligand design

The ligand-mapping MD trajectory structure with the shortest distance between Phe482 and the alternatively-bound benzene was used for ligand design. By superimposing this structure on the crystal structure of a PBD peptide complex (PBD code 3P37), a chimeric peptide was designed by replacing the N-terminal FDP residues of the peptide in the crystal structure with a 3-phenylpropanoyl moiety such that the phenyl ring approximately occupies the position of the bound benzene. The PBD trajectory structure complexed with the chimeric peptide was used as the initial structure for MD simulation using the same protocol as described above, except that the production run was performed for 20 ns.

Atomic charges for benzene and the 3-phenylpropanoyl fragment were derived using the R.E.D. Server,[17] which fits restrained electrostatic potential (RESP) charges[18] to a molecular electrostatic potential (MEP) computed by the Gaussian 09 program[19] at the HF/6-31G* theory level.

Trajectory analysis

Non-hydrogen atoms of the 7 pocket residues (Val415, Tyr417, Tyr421, Leu478, Tyr481, Phe482 and Tyr485) were clustered using the MMTSB toolset.[20] The ART-2 algorithm[21-22] was used for RMSD-based clustering. Suitable cutoff radii (1.6 Å for the unliganded simulations and 1.4 Å for the ligand-mapping simulations) were empirically chosen to produce clusters containing as many centroid conformations corresponding to crystal structure conformations as possible. Clusters related by a 180° flip of Phe and Tyr aromatic rings about χ_2 were identified and combined. PyMOL[23] and Visual Molecular Dynamics (VMD)[24] were used for visualizing and generating figures.

Benzene occupancy grids were generated by using the ptraj module of AMBER 11 to bin carbon atoms of benzene molecules into 1 Å × 1 Å × 1 Å grid cells. A grid was generated for each of the three benzene binding modes using 100 snapshots exhibiting the relevant binding mode from a ligand-mapping MD trajectory. An isocontour value of 7 was used for visualization of benzene occupancy in the grids.

Experimental validation

Protein preparation

Truncated construct of PBD of human Plk1 (residues 371-594) used for crystallization and full-length His_6-PBD (residues 345-603) construct were expressed in *E. coli* and purified in two chromatographic steps according to a previously reported procedure.[2]

Chimeric peptide preparation

Chimeric peptide was prepared by manual solid phase peptide synthesis (SPPS) using an Fmoc protection group strategy on a NovaBioChem Rink Amide AM resin (100-200 mesh). Crude peptide was purified by reverse-phase HPLC on a Gilson GX-271 equipped with a Gilson 171 diode array detector using a Polaris C8-A (5 μm; 4.6 x 300 mm (analytical), 21.2 x 300 mm (preparative); Varian, inc.) column at 1 mL/min (analytical) or 21 mL/min (preparative) using a linear gradient of 5 % to 60 % B over 30 min. The solvent system used was A (0.1 % (v/v) TFA in H_2O) and B (0.1 % (v/v) TFA in acetonitrile). Peptide identity was confirmed by MALDI-TOF-MS (ABI 4700 Proteomics Analyzer, Applied Biosystems) and amino acid analysis (PNAC facility, Department of Biochemistry, University of Cambridge).

Structure determination

PBD-peptide complex was generated by mixing the protein with the peptide in the molar ratio of 1:1.2. The complex was crystallized in a sitting drop setup by mixing a solution of complex concentrated to 10-15 mg/mL with the well solution containing 0.1 M HEPES (pH 7.5), 0.1 M sodium azide and 22% PEG 4000 in a 1:1 (v/v) ratio.

Experimental diffraction data were collected in-house using an X8 Proteum diffraction system equipped with MICROSTAR rotating anode x-ray generator, Helios MX optics and Platinum135 CCD detector. Data were processed with PROTEUM software package and the structure was solved by molecular replacement using PHASER[25] from the CCP4 program suite and the 3P2Z structure (chain A) as the search model. The model was manually rebuilt with Coot[26] and refined using Refmac.[27] The summary of data collection and refinement is shown in Table S1.

Isothermal titration calorimetry

ITC experiments were performed using the ITC200 instrument (Microcal Inc. – GE Healthcare) at 25 °C. His_6-PBD345-603 was loaded into the ITC cell at concentration of 34 μM. Ligand was dissolved in the same buffer to the concentration of 500 μM. 36 injections of 1.0 μL in volume were done over a period of 50 min. Data was fitted to single binding site model using the Origin software package provided by the manufacturer. Titration curve and thermodynamic parameters of binding are shown in Figure S5.

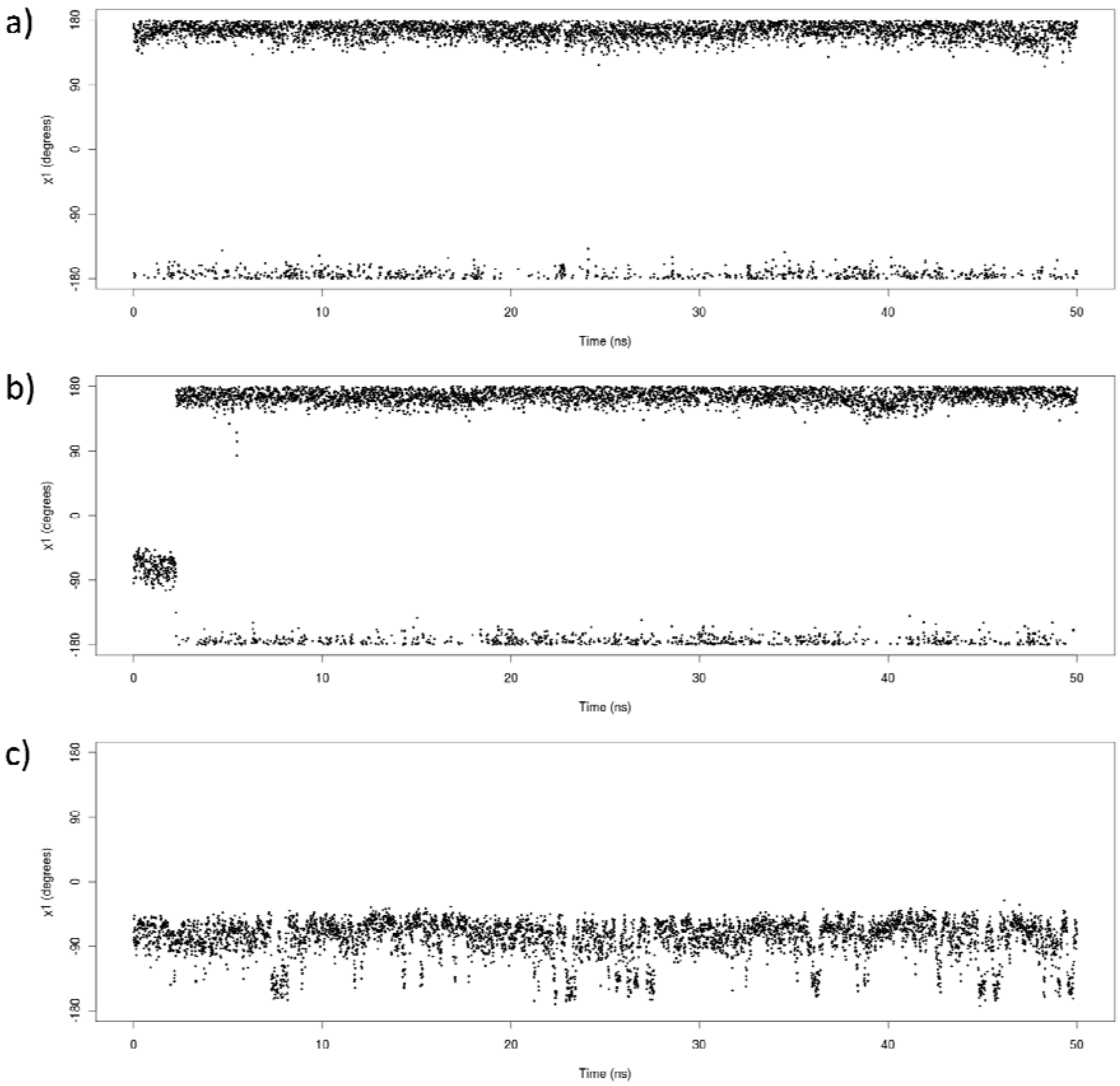

Figure S1. χ_1 dihedral angle of Y481 during MD simulations of (a) unliganded PBD starting from closed second pocket (b) unliganded PBD starting from open pocket. (c) PBD complexed with FDPPLHSpTA peptide.

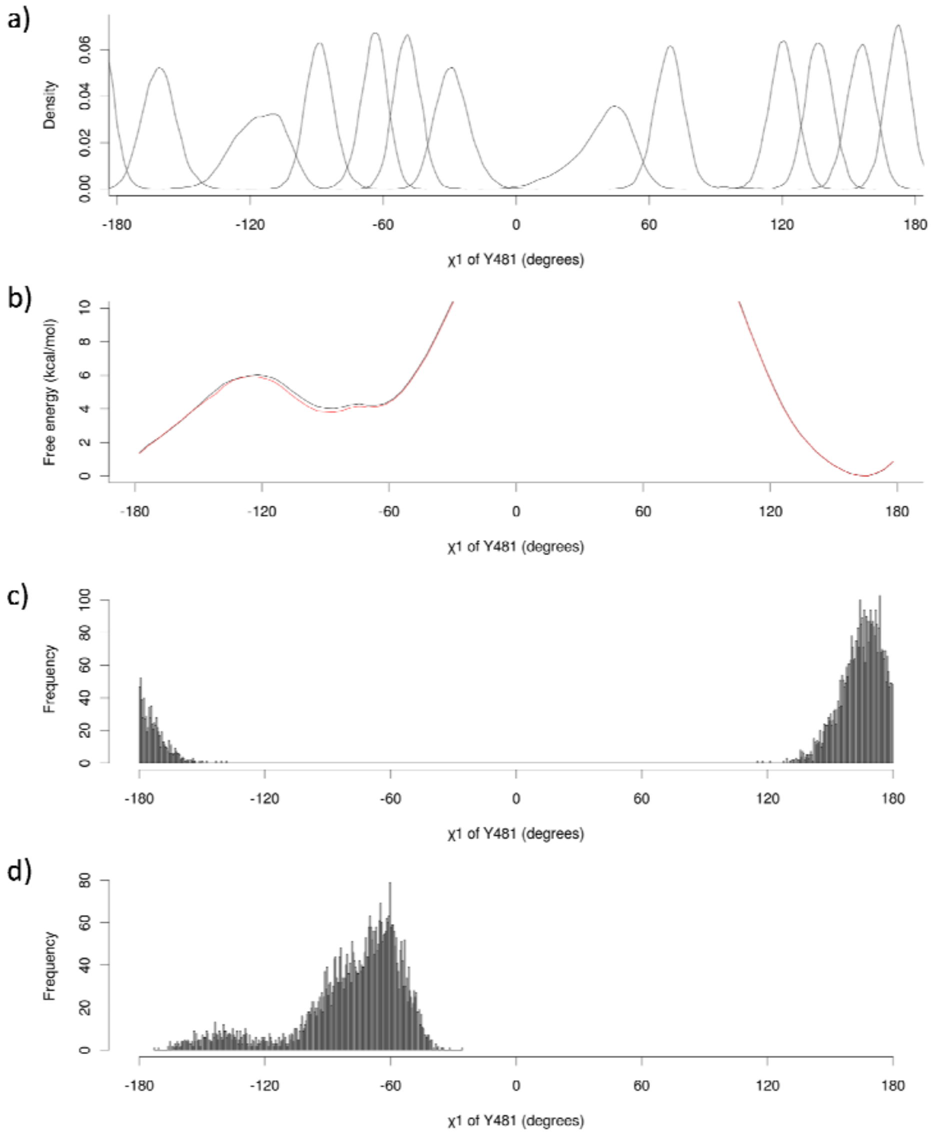

Figure S2. Umbrella sampling analysis. (a) Distribution of Y481 χ_1 values in umbrella sampling simulations. (b) Free energy profiles for χ_1 of Y481 obtained from umbrella sampling. High-energy regions which contribute little to equilibrium populations have been omitted. The profiles generated with data from 1-2 ns (red) and 1-3 ns (black) are very similar, indicating convergence of the sampling. (c) Distribution of Y481 χ_1 values in unbiased trajectories of unliganded PBD with closed pocket. (d) Distribution of Y481 χ_1 values in unbiased trajectories of complexed PBD with open pocket.

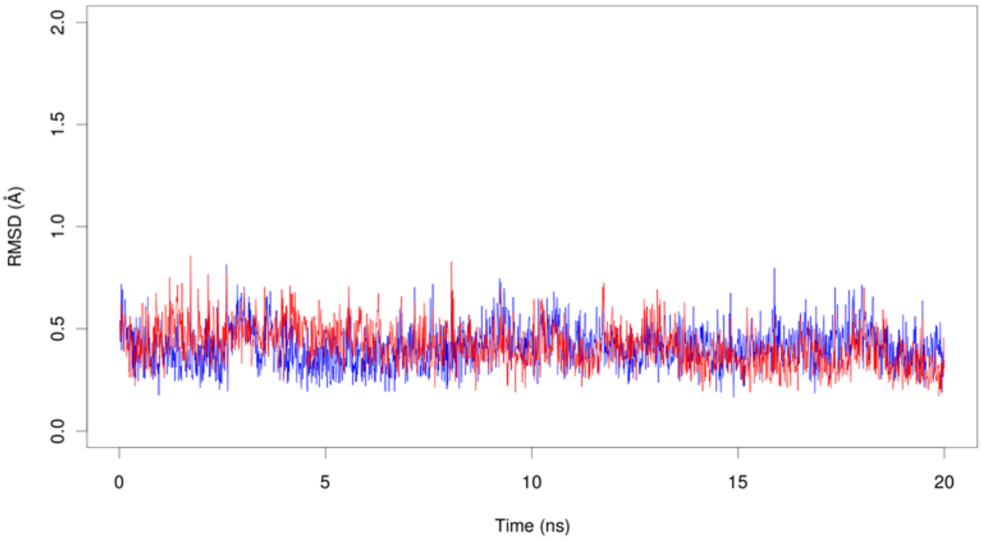

Figure S3. Backbone root mean square deviation (RMSD) of chimeric peptide in complex with Plk1 from the minimized designed structure used to initialize the MD simulation (blue) and from the crystal structure (red).

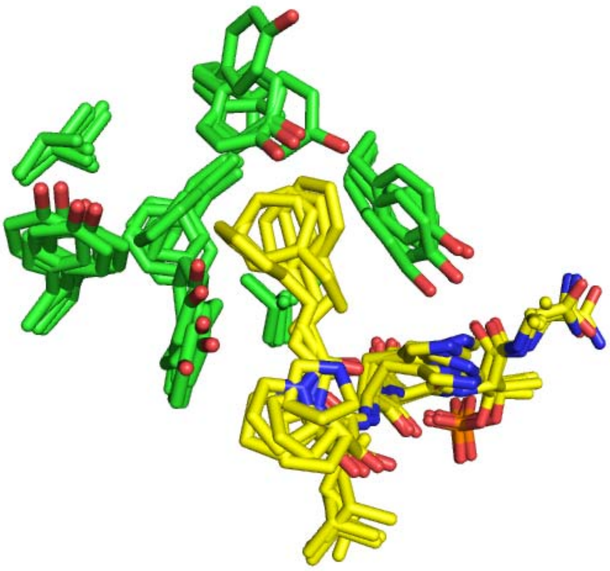

Figure S4. Superposition of five MD snapshots of designed chimeric peptide bound at PBD hydrophobic binding site.

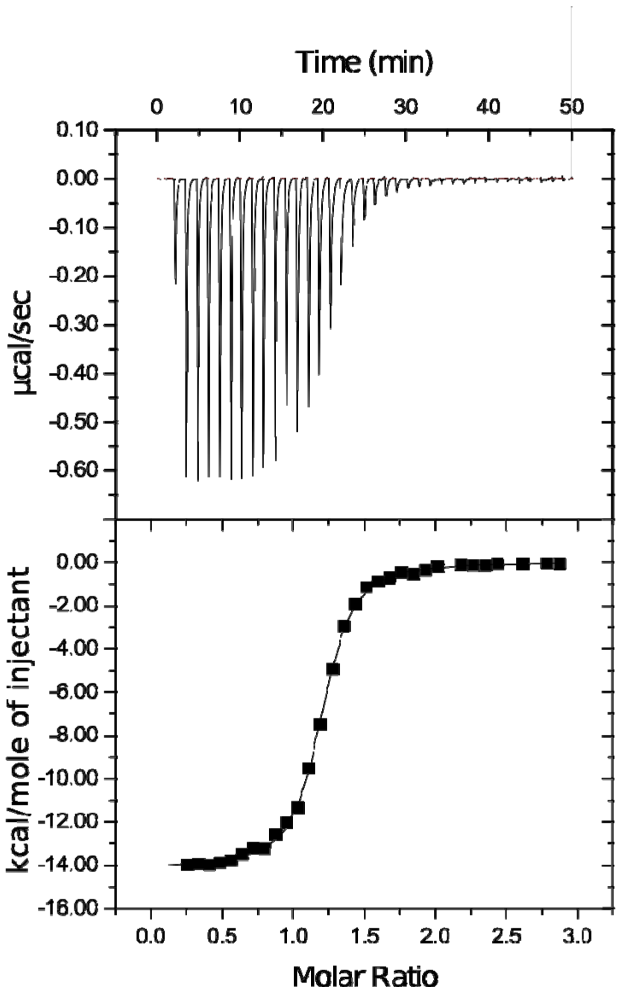

ΔH (kcal/mol)	-14.1 ± 0.1
K_D (nM)	330 ± 14
n	1.17

Figure S5. ITC titration curve for the binding of chimeric peptide and its fit to a single site binding model.

Table S1. Data collection and refinement statistics for PBD-chimeric peptide complex.

**PDB Id**	4E67
**Data collection**	
X-ray source	MICROSTAR (in house)
Wavelength (Å)	1.5406
Space group	$P2_1$
Cell dimensions	
a, b, c (Å)	35.49 50.91 57.91
α, β, γ (°)	90.0 101.0 90.0
Resolution	56.84 – 2.10
(high res shell)	(2.20 – 2.10)
Rsym	5.5 (26.3)
I/σI	14.01 (2.14)
Completeness (%)	98.6 (90.7)
Redundancy	2.85 (1.34)
No. reflections	11.821
**Refinement**	
Rwork/Rfree	21.9 (26.7)
PBD molecules in the asymmetric unit	1
No. atoms	
Protein*	1774
Water	76
RMS deviations	
Bond lengths (Å)	0.007
Bond angles (°)	1.0
**Model quality**	
B-factors	
Protein*	20.0
Water	25.5
Ramachandran	
Favoured	98.1
Allowed	1.9
Outlier	0.0

* Chimeric phosphopeptide was treated as protein

References:

[1] S. M. Yun, T. Moulaei, D. Lim, J. K. Bang, J. E. Park, S. R. Shenoy, F. Liu, Y. H. Kang, C. Z. Liao, N. K. Soung, S. Lee, D. Y. Yoon, Y. Lim, D. H. Lee, A. Otaka, E. Appella, J. B. McMahon, M. C. Nicklaus, T. R. Burke, M. B. Yaffe, A. Wlodawer, K. S. Lee, *Nat. Struct. Mol. Biol.* **2009**, *16*, 876-882.

[2] P. Sledz, C. J. Stubbs, S. Lang, Y. Q. Yang, G. J. McKenzie, A. R. Venkitaraman, M. Hyvonen, C. Abell, *Angew. Chem. Int. Ed.* **2011**, *50*, 4003-4006.

[3] T. J. Dolinsky, P. Czodrowski, H. Li, J. E. Nielsen, J. H. Jensen, G. Klebe, N. A. Baker, *Nucleic Acids Res.* **2007**, *35*, W522-W525.

[4] D. A. Case, T. A. Darden, T. E. Cheatham, C. L. Simmerling, J. Wang, R. E. Duke, R. Luo, R. C. Walker, W. Zhang, K. M. Merz, B. Roberts, B. Wang, S. Hayik, A. Roitberg, G. Seabra, I. Kolossvái, K. F. Wong, F. Paesani, J. Vanicek, J. Liu, X. Wu, S. R. Brozell, T. Steinbrecher, H. Gohlke, Q. Cai, X. Ye, J. Wang, M.-J. Hsieh, G. Cui, D. R. Roe, D. H. Mathews, M. G. Seetin, C. Sagui, V. Babin, T. Luchko, S. Gusarov, A. Kovalenko, P. A. Kollman, University of California, San Francisco, **2010**.

[5] W. L. Jorgensen, J. Chandrasekhar, J. D. Madura, R. W. Impey, M. L. Klein, *J. Chem. Phys.* **1983**, *79*, 926-935.

[6] V. Hornak, R. Abel, A. Okur, B. Strockbine, A. Roitberg, C. Simmerling, *Proteins: Struct. Funct. Bioinform.* **2006**, *65*, 712-725.

[7] K. Lindorff-Larsen, S. Piana, K. Palmo, P. Maragakis, J. L. Klepeis, R. O. Dror, D. E. Shaw, *Proteins: Struct. Funct. Bioinform.* **2010**, *78*, 1950-1958.

[8] J. M. Wang, R. M. Wolf, J. W. Caldwell, P. A. Kollman, D. A. Case, *J. Comput. Chem.* **2004**, *25*, 1157-1174.

[9] N. Homeyer, A. H. C. Horn, H. Lanig, H. Sticht, *J. Mol. Model.* **2006**, *12*, 281-289.

[10] J. P. Ryckaert, G. Ciccotti, H. J. C. Berendsen, *JCoPh* **1977**, *23*, 327-341.

[11] T. Darden, D. York, L. Pedersen, *J. Chem. Phys.* **1993**, *98*, 10089-10092.

[12] J. A. Izaguirre, D. P. Catarello, J. M. Wozniak, R. D. Skeel, *J. Chem. Phys.* **2001**, *114*, 2090-2098.

[13] H. J. C. Berendsen, J. P. M. Postma, W. F. Vangunsteren, A. Dinola, J. R. Haak, *J. Chem. Phys.* **1984**, *81*, 3684-3690.

[14] G. M. Torrie, J. P. Valleau, *JCoPh* **1977**, *23*, 187-199.

[15] S. Kumar, D. Bouzida, R. H. Swendsen, P. A. Kollman, J. M. Rosenberg, *J. Comput. Chem.* **1992**, *13*, 1011-1021.

[16] L. Martinez, R. Andrade, E. G. Birgin, J. M. Martinez, *J. Comput. Chem.* **2009**, *30*, 2157-2164.

[17] E. Vanquelef, S. Simon, G. Marquant, E. Garcia, G. Klimerak, J. C. Delepine, P. Cieplak, F.-Y. Dupradeau, *Nucleic Acids Res.* **2011**, *39*, W511-W517.

[18] W. D. Cornell, P. Cieplak, C. I. Bayly, P. A. Kollman, *J. Am. Chem. Soc.* **1993**, *115*, 9620-9631.

[19] M. J. Frisch, G. W. Trucks, H. B. Schlegel, G. E. Scuseria, M. A. Robb, J. R. Cheeseman, G. Scalmani, V. Barone, B. Mennucci, G. A. Petersson, H. Nakatsuji, M. Caricato, X. Li, H. P. Hratchian, A. F. Izmaylov, J. Bloino, G. Zheng, J. L. Sonnenberg, M. Hada, M. Ehara, K. Toyota, R. Fukuda, J. Hasegawa, M. Ishida, T. Nakajima, Y. Honda, O. Kitao, H. Nakai, T. Vreven, J. Montgomery, J. A., J. E. Peralta, F. Ogliaro, M. Bearpark, J. J. Heyd, E. Brothers, K. N. Kudin, V. N. Staroverov, R. Kobayashi, J. Normand, K. Raghavachari, A. Rendell, J. C. Burant, S. S. Iyengar, J. Tomasi, M. Cossi, N. Rega, N. J. Millam, M. Klene, J. E. Knox, J. B. Cross, V. Bakken, C. Adamo, J. Jaramillo, R. Gomperts, R. E. Stratmann, O. Yazyev, A. J. Austin, R. Cammi, C. Pomelli, J. W.

Ochterski, R. L. Martin, K. Morokuma, V. G. Zakrzewski, G. A. Voth, P. Salvador, J. J. Dannenberg, S. Dapprich, A. D. Daniels, Ö. Farkas, J. B. Foresman, J. V. Ortiz, J. Cioslowski, D. J. Fox, Gaussian, Inc., Wallingford CT, **2009**.

[20] M. Feig, J. Karanicolas, C. L. Brooks, *J. Mol. Graphics Model.* **2004**, *22*, 377-395.

[21] G. A. Carpenter, S. Grossberg, *Appl. Opt.* **1987**, *26*, 4919-4930.

[22] M. E. Karpen, D. J. Tobias, C. L. Brooks, *Biochemistry (Mosc).* **1993**, *32*, 412-420.

[23] W. L. DeLano, DeLano Scientific, San Carlos, CA, USA, **2002**.

[24] W. Humphrey, A. Dalke, K. Schulten, *J. Mol. Graphics* **1996**, *14*, 33-38.

[25] A. J. McCoy, *Acta Crystallogr. Sect. D. Biol. Crystallogr.* **2007**, *63*, 32-41.

[26] P. Emsley, K. Cowtan, *Acta Crystallogr. Sect. D. Biol. Crystallogr.* **2004**, *60*, 2126-2132.

[27] G. N. Murshudov, A. A. Vagin, E. J. Dodson, *Acta Crystallogr. Sect. D. Biol. Crystallogr.* **1997**, *53*, 240-255.
